# Donovanosis^⋆^^⋆⋆^

**DOI:** 10.1016/j.abd.2020.07.002

**Published:** 2020-09-17

**Authors:** Walter Belda Junior

**Affiliations:** Department of Dermatology, Faculty of Medicine, Universidade de São Paulo, São Paulo, SP, Brazil

**Keywords:** Granuloma inguinale, Sexually transmitted diseases, Sexually transmitted diseases, bacterial

## Abstract

Donovanosis is a chronic, progressive, and indolent bacterial disease that affects the skin and mucous membranes of the genital and perigenital regions, often associated with sexual transmission, and with low infectivity. The malignant transformation of donovanosis lesions occurs exceptionally, as is usually seen in long-term ulcerations.

## Introduction

Donovanosis is a chronic, progressive, and indolent bacterial disease that affects the skin and mucous membranes of the genital and perigenital regions, often associated with sexual transmission, and with low infectivity. The malignant transformation of donovanosis lesions occurs exceptionally, as is usually seen in long-term ulcerations.^1^, ^2^, ^3^

## Historic

It was first described by McLeod, in Calcutta, India, in 1882.^4^ In 1898, Manson reported lesions in black individuals from East India and suggested the possibility of cases in other tropical countries. Manson presented a thorough clinical description and termed the disease “pudendal ulcerative granuloma.” The term “donovanosis” originates from the description of the etiological agent of granuloma inguinale made in 1905 by Donovan, when working in a hospital in Madras. Donovan detailed the presence of intracellular inclusions in macrophages after a morphological study with special stains of material extracted from lesions of his patients, which he believed to be caused by a protozoan.^5^ This concept persisted for three decades, given the inability to culture the material in different types of culture media. These intracellular inclusions were later termed “Donovan bodies.” At the time, treatment with antimonial was suggested. Antimonials were already being used to treat kala-azar – Leishman described the intracellular bodies in liver and spleen cells in, 1903, and a similar observation was later made by Donovan. This sequence of discoveries explains the term “Leishman-Donovan bodies.” Donovan bodies in granuloma inguinale were then considered protozoa, analogous to Leishman bodies and, therefore, the same treatment was indicated.^6^

In 1913, Aragão and Viana introduced intravenous emetic tartar treatment, the first effective drug in the treatment of donovanosis.^7^ In 1926, McIntosh reported the transmission of the disease in volunteers through subcutaneous transplantation of infected tissue.^8^ In 1931, Monbreun and Goodpasture tried to inoculate material rich in Donovan bodies, without success. They established, however, that the microorganism causing the disease was not a protozoan. They described a Gram-negative bacillus that grew in a chorioallantoic membrane filtrate of chicken embryos, in capsulated and non-capsulated forms. In 1939, Greenblat et al. reproduced the disease in four volunteers using material from pseudobuboes, but failed to grow any organism in the chorioallantoic membrane of chicken embryos.^9^ In 1943, Anderson managed to isolate the microorganism in a yolk sac from embryonated eggs, demonstrating that it was a Gram-negative, immobile bacterium, found in encapsulated and non-encapsulated forms, which would be capable of producing antigen that resulted in positive reactions when injected intradermally in patients. Believing this to be the causal agent of the disease, the author proposed the creation of a new species: *Donovania granulomatis*. The term donovanosis was proposed by Marmell and Santora in 1950 in honor of Donovan, and was accepted by the scientific community.^10^ Based on a sample of over 800 cases, in 1987 Jardim created the first clinical classification of the disease, which has been adopted in the medical literature to the present day.^11^

Many synonyms for donovanosis can be found in the literature, such as phagedenic disease of the genitals, contagious granuloma, sclerosing granuloma, infectious granuloma, venereal granuloma, and the fifth venereal disease. These terms highlighted the clinical and epidemiological aspects of the disease.

Much of what is known about donovanosis derived from studies carried out during the first half of the last century; few studies were conducted on the subject between the mid-1960s and 1990s. Since 1990, there has been an increase in scientific interest in this sexually transmitted infection (STI); much of this interest has arisen from the association between genital ulcers and HIV transmission.

## Etiology

The agent of donovanosis is a Gram-negative, facultative aerobic coccobacillus, which stains with greater intensity in the extremities than in the center, whether or not it is encapsulated, and which is intracytoplasmic and immobile. As a rounded cocus, it measures 0.02 µm to 0.2 µm; in the bacillary form, it measures 1.0 µm–2.5 µm in length.^12^ In the lesions, these microorganisms are found inside macrophages in the form of small oval bodies, called Donovan bodies. They are stained with relative ease by Giemsa, Leishman, or Wright staining. They were initially classified by Aragão and Vianna as *Calymmatobacterium granulomatis*; however, they were very similar to *Klebsiella* spp., a similarity confirmed by Rake's studies based on serological crossing and, later, by ultrastructural differences.^7^, ^13^ In 1999, based on molecular aspects, through which a total of 2089 base pairs of the 165rRNA and phoE genes were sequenced, Carter et al. demonstrated that *C. granulomatis* was more than 90% similar to *K. pneumoniae* and *K. rhinoscleromatis*, proposing then the reclassification of the agent to *Klebsiella granulomatis comb. nov*., which remains to date.^14^, ^15^

## Epidemiology

Although the disease has been described for more than a century, it is often overlooked due to its geographical distribution and low incidence. Therefore, it is not surprising that there are few published data on its incidence, even in endemic areas.

Donovanosis is more common in blacks and individuals with poor hygiene, being endemic in tropical and subtropical countries such as Papua New Guinea, South Africa, India, Indonesia, Australia, the Caribbean, Argentina, French Guiana, and Brazil. However, it is likely that this distribution is more related to socioeconomic conditions and living conditions than to racial or geographical factors.^16^, ^17^ However, in a study by O'Farrel in South Africa, with a rapid test for the diagnosis of donovanosis, there was an increase from 312 cases in 1988 to 2385 cases in 1995, suggesting that the diagnosis of the disease has been underestimated in several areas where it is prevalent.^17^ Additional increases were reported in 1996 and 1997, with 2733 and 3153 cases, respectively.^18^ It is not clear whether these data reflect a genuine increase in prevalence or a higher rate of clinical suspicion. Morrone et al. suggested that, even in developed countries, doctors have difficulty in identifying cases of donovanosis due to their lack of experience with tropical diseases or because the disease is treated and accidentally cured by the use of self-administered antibiotics.^19^

Despite the worldwide decrease in cases of bacterial STIs and an increase in viral STIs, there is evidence to suggest a higher prevalence of unprotected sex after the introduction of antiviral therapy for HIV, which has been increasing the incidence of STIs in several industrialized and developing countries.^20^ Syndromic diagnosis and treatment in various areas of the world has made it even more difficult to statistically assess the prevalence of STIs, which is exemplified by O’Farrel’s observations in South Africa.^21^ All these factors explain why the real incidence of the disease is not known; however, there is a consensus in the literature that the incidence of donovanosis has been sharply decreasing and that it can be classified as a sporadic disease.

Its incidence varies between the sexes in different studies, but there appears to be no gender predilection. It affects almost exclusively adults aged 20–40 years, a period of greater sexual activity; no congenital infections have been reported. However, there are reports of cases in infants and newborns, generally associated with contact with infected adults and not necessarily due to sexual abuse. There are few reports of perinatal donovanosis transmission, but the apparent predilection for otorhinolaryngological structures is noteworthy. Govender et al. reported two cases in which young children were diagnosed with donovanosis without a history of sexual abuse.^22^ An eight-month-old child had a tumor and seropurulent discharge from the right ear. She was initially treated with antibiotics and surgical resection, but the case was not followed-up for nine months. In a latter appointment, it was observed that the condition had worsened, with the presence of an abscess in the right temporal lobe. The patient underwent craniectomy for drainage of the abscess, as well as mastoidectomy. The tissue removed during surgery was analyzed, confirming the diagnosis of donovanosis. Questioning of the parents indicated no signs or symptoms of sexual abuse. In that same study, another case of a 5-month-old child with purulent discharge from the left ear, associated with paralysis of the VII cranial nerve, tumor in the external auditory canal, and friable retroauricular abscess was reported. Samples obtained from tissue during surgical resection were also conclusive for donovanosis. The mother's gynecological examination revealed a cervical lesion confirmed by histological examination to be a granuloma inguinale. No signs or symptoms of sexual abuse were observed. Scott et al. also reported a case of a 5-month-old child with purulent discharge from the ear and tympanic perforation, whose mother had untreated cervical donovanosis.^23^ Other authors have also reported the occurrence of donovanosis in children with weeks of life who developed otorhinolaryngological and skin lesions due to donovanosis, without history of sexual abuse, thus emphasizing the possibility of transmission during childbirth.^24^

The concomitance between donovanosis and STIs, including HIV, is well documented in the literature. As most cases of donovanosis presents clinically as a genital ulcer, the carrier has a 4.7 times increased risk of contracting HIV.^3^

As for its form of transmission, there is an interesting discussion in the literature if donovanosis is really an STI; there are two lines of thought regarding its transmission. The arguments that support the hypothesis of sexual transmission include:^3^, ^21^, ^25^

Higher incidence in the age group of greater sexual activity.

Lesions found in the internal genitalia, such as uterine cervix, without other manifestations.

Presence of anal lesions associated with the practice of anal sex.

Presence of external genital or near-genital lesions in most cases.

Concomitant STI.

Sexual contact with sex workers.

High prevalence in sexual partners in some studies.

In turn, arguments that these facts would not be sufficient to establish sexual transmission as definitive, supporting the non-sexual transmission of the disease, include:^16^, ^21^

Occurrence in children and adults without sexual activity.

Relative rarity in sex workers.

Unclear incubation period.

Low rates of infection among sexual partners in some geographic areas.

Occurrence of non-genital lesions in homosexuals and heterosexuals.

Another argument contrary to sexual transmission was the isolation of microorganisms from the feces of a patient with donovanosis, morphologically similar to *C. granulomatis*.^26^ From this material, it was prepared an antigen that produced a positive reaction with serum from patients with donovanosis. The author concluded that these results reinforced the hypothesis that a saprophytic organism, existing in the feces, was the etiological agent of donovanosis. Contamination would occur through anal or vaginal intercourse when there was contamination with feces. This would also explain the pediatric cases, with no previous history of sexual abuse, and cases with lesions on the face and lower limbs without concomitant genital involvement.

Despite the possibility of transmission through direct inoculation in some cases, donovanosis is currently considered primarily a STI, in which the main clinical manifestations occur in the genital region, with extragenital lesions occurring in 6% of cases. In favor of this argument is its higher incidence in sexually active groups, the predominant location in the genital areas (80%–100% of cases), and the frequent association of donovanosis, HIV, and other STIs in the same patient.^27^ Nonetheless, it is important to note that when sexual intercourse is considered as a mode of transmission, the infectivity of granuloma inguinale is low. From a practical standpoint, it is not possible to rule out the diagnosis of donovanosis in a patient with this diagnostic hypothesis considering only the absence of the disease in their sexual partners in the last six months.^28^, ^29^

## Clinical manifestations

The incubation period is controversial and not well established in the literature; some studies indicate periods from two weeks to one month, others from 42 to 50 days, and experimental inoculations in human volunteers produced lesions after a period of 21 days.^30^, ^31^, ^32^ Genitals are affected in 90% of cases and the inguinal region in 10%. Extragenital involvement are observed in 6% of cases.^33^ In general, the regions most affected in men are the coronal groove, the balanoprepucial area, and the anus. In women, the areas most commonly affected are the labia minora, vaginal furcula, and, occasionally, the cervix and upper genital tract, where they can simulate carcinomas. In the initial stage of donovanosis, a papular lesion or subcutaneous nodule evolves to ulceration with a papular surface or subcutaneous nodule at the inoculation site, ulceration of an erythematous, shiny, friable, and slow-growing surface. In general, the lesions are not painful and there is no inguinal adenopathy; however, the initial lesions in the inguinal topography simulate a bulbo (pseudobulbo). As the disease progresses, the manifestations are directly linked to the effectiveness of the host’s tissue response, giving rise to localized forms in the large majority of patients and occasionally to visceral lesions by hematogenous dissemination.^3^, ^27^

The observation of the polymorphism of these manifestations led Jardim to propose a clinical classification that comprises the following forms:^11^

1. Genital and perigenital:

1.1 Ulcerative;

1.1.1 With hypertrophic edges;

1.1.2 With flat edges.

1.2. – Ulcerative-vegetative;

1.3 Vegetative;

1.4 Elephantiasis.

2. Extragenital.

**Figure 1:**
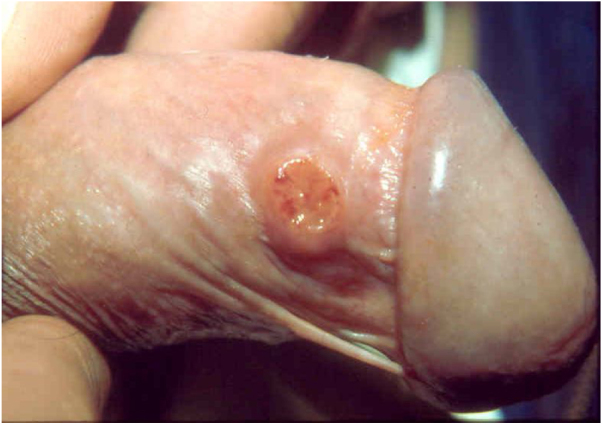
Nodular-ulcerated lesion located in the balanoprepucial groove.

**Figure 2:**
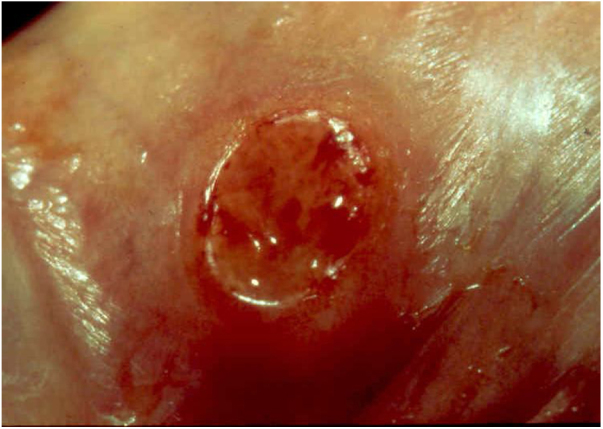
Ulcerated lesion **located in the balanoprepucial groove**, with granular background and bleeding. .

**Figure 3:**
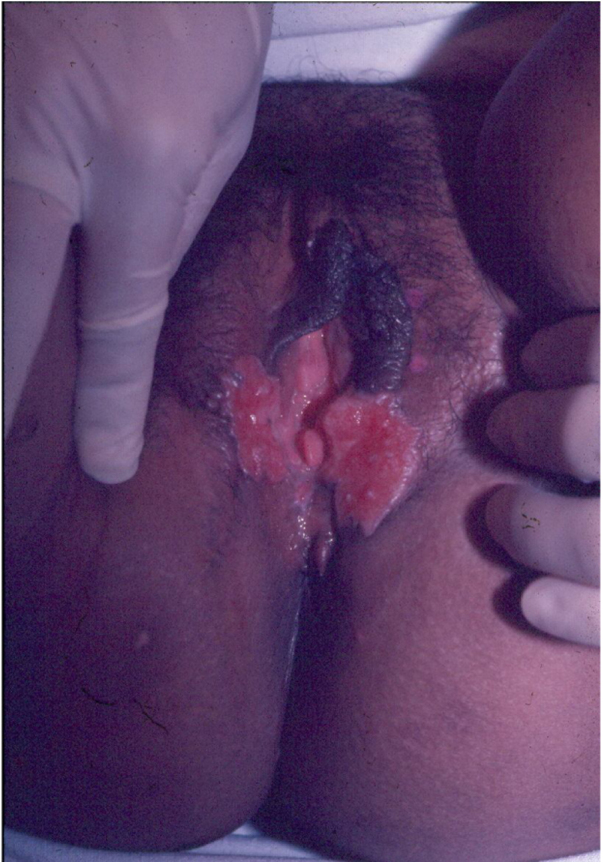
Extensive ulcerative-vegetative lesions, with mirror image (Image credits: Professor Sinésio Talhari and Professor Adele Benzaquem).

**Figure 4:**
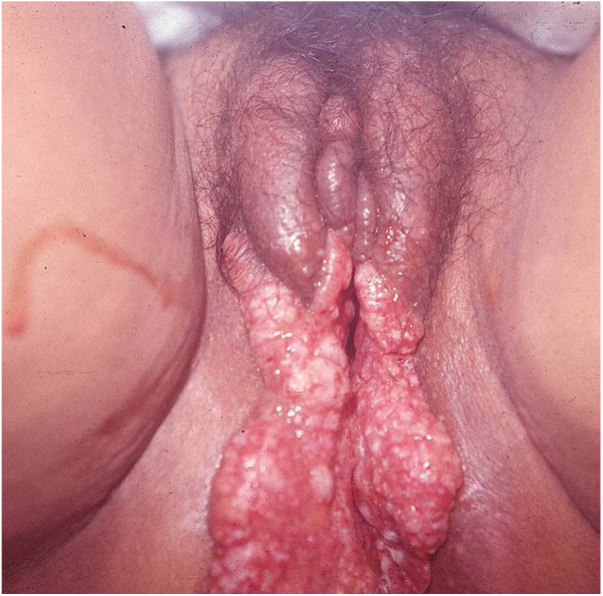
Lymphedema of the labia majora and minora with extensive, mirror-image vegetative ulcers (Image credits: Professor Sinésio Talhari and Professor Adele Benzaquem).

Ulcerative forms may be lenticular in size, have everted edges, and present a mild inflammatory reaction. They grow slowly, becoming well defined, with a granular background, have a centrifugal and serpiginous character, and are covered with a thick and fetid exudate (Fig. 1). They present abundant secretion and bleed easily (Fig. 2). Their edges have a variable aspect, firm consistency, and are smooth; they are not undermined or excavated; they can be flat or hypertrophic. The edges may eventually become raised, clearly defining the lesion and sometimes assuming a carcinomatous aspect.^27^ As these lesions grow, due to centrifugal extension, the central part sometimes presents a regressive scarring process. The lesions can expand by contiguity, in a linear shape; they are frequent linear in the skin folds and often present a mirror configuration. They are preferably located in folds and can become very extensive (Figure 3, Figure 4). Eventually, secondary bacterial infection may develop in the lesions, in addition to the possibility of coexistence with other sexually transmitted pathogens.^21^, ^34^, ^35^ In the ulcerative-vegetative form, the most frequently observed, the abundant granulation tissue at the bottom of the ulcerated lesion goes beyond the lesional contour, bleeding very easily. In turn, the vegetative forms are characterized by lesions without secretion that usually present with small dimensions and well-defined borders. These forms are uncommon.^27^

Elephantiasis manifestations (esthiomene) almost always occur after long-lasting ulcerated forms. They are consequent to lymphatic involvement (Fig. 4). With evolution, the lesions heal with intense fibrosis, causing destruction of the lymphatic pathways. These forms are mainly observed in the female genitalia, being rare in male patients. This is the most frequent complication of donovanosis. There are also reports of the development of squamous cell carcinomas of the vulva after a long course of the disease.^2^, ^36^, ^37^

**Figure 5:**
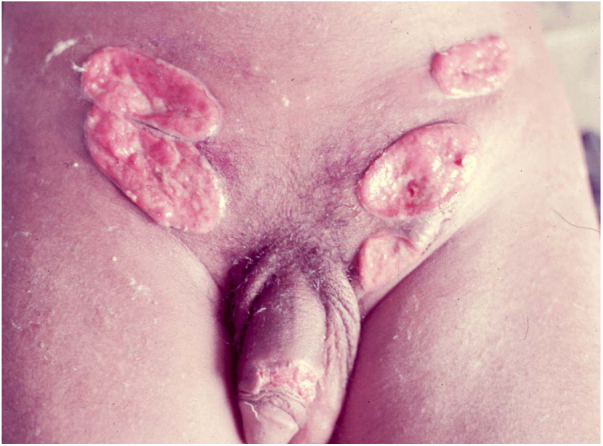
Ulcerated lesion lesion on the penis and ulcerative vegetative lesions (Image credits: Professor Sinésio Talhari ).

Extra-genital locations (Fig. 5) may be a consequence of sexual practices or may be acquired by direct inoculation in areas with a solution of continuity, particularly through contaminated fingers, which is possibly the most frequent form. These infrequent locations include the lips, gums, jaw, palate, larynx, pharynx, neck, nose, ophthalmic region, armpits, chest, abdomen, scalp, joints, and bones, particularly the tibia.^16^, ^17^, ^38^, ^39^ There may be involvement of the lungs, intestine, spleen, liver, uterus, and ovaries, especially in immunocompromised patients. The clinical picture in these patients leads to changes in general condition, such as fever, anemia, night sweats, weight loss, and severe toxemia, putting the patient’s life at serious risk, as the diagnosis is rarely suspected. A classic case is that of donovanosis in women who have cervical lesions and undergo hematogenous dissemination to lumbar vertebrae, which leads to death.^3^ In these cases with fatal evolution, the diagnosis of disseminated donovanosis is usually made only at necropsy.

In patients with AIDS, due to the severe immunological depression that can occur, donovanosis can evolve with atypical clinical manifestations, with the appearance of new lesions or the expansion of those pre-existing, as well as persistence of bacteriological positivity, despite adequate treatment.^17^, ^40^

## Donovanosis and pregnancy

The possibility of depression of the cell-mediated immune response during pregnancy can favor infections by intracellular agents, such as donovanosis. The disease has a more aggressive course during pregnancy. In prenatal care, the hypothesis of donovanosis should be considered in cases of atypical lesions.^17^, ^41^ If, eventually, the internal genitalia is affected, the risk of hematogenous dissemination is greater, which can lead to serious complications during pregnancy and childbirth. Eventual surgical reconstruction of chronic injuries should be postponed until the end of the pregnancy. When there is a risk or the possibility of perineal laceration, or even the possible contamination of the fetus during vaginal delivery, cesarean section should be the first option.

## Donovanosis/HIV coinfection

The presence of genital ulcerations increases the risk of HIV transmission by up to 4.7 times after unprotected sexual exposure to a person infected with the virus. There are few studies on the relationship of the effects of HIV infection on donovanosis. However, as ulcers bleed very easily, it is understood that this disease is a large risk factor for the acquisition of HIV, a possibility that increases with the long duration of injuries. In areas where donovanosis is endemic, the prevalence of HIV has increased significantly since the beginning of its epidemic. This epidemiological aspect was observed in Durban, South Africa. This increase in HIV transmission has also been associated with the introduction of the syndromic approach for the treatment of STIs.^17^, ^21^, ^40^ HIV tests, guidance, and counseling should be considered for all cases of donovanosis. Co-infection with HIV usually causes persistent ulcers for longer periods and requires longer treatment when compared with HIV-negative donovasis patients.^37^, ^41^, ^42^

## Diagnosis

The diagnosis must be made taking into account the clinical aspect of the lesions, but it depends primarily on the demonstration of Donovan bodies in material obtained from the lesions, using cytological preparations and histological sections for confirmation.^3^, ^28^ The presence of cells with Donovan bodies is pathognomonic for the infection.

## Cytodiagnosis

**Figure 6:**
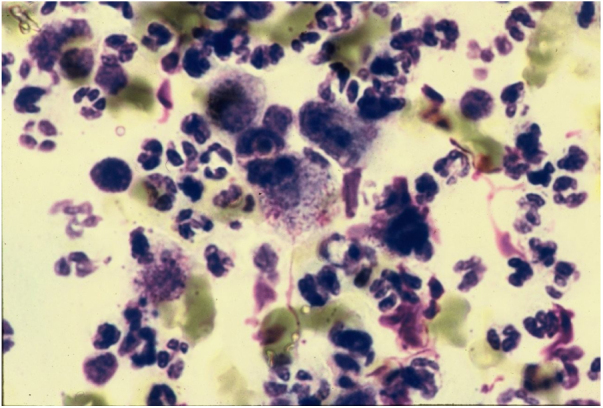
Histiocytes with Donovan bodies. Giemsa staining.

Direct microscopy is the fastest, most economical, and most reliable diagnostic method. The material should preferably be collected from areas of active granulation that do not show signs of secondary infection. The smears should be preferably collected from part of the fragment excised for anatomopathological examination, which should be pressed against a glass slide and then stained with Giemsa, Leishman or Wright staining (Fig. 6).^43^

## Histopathology

**Figure 7:**
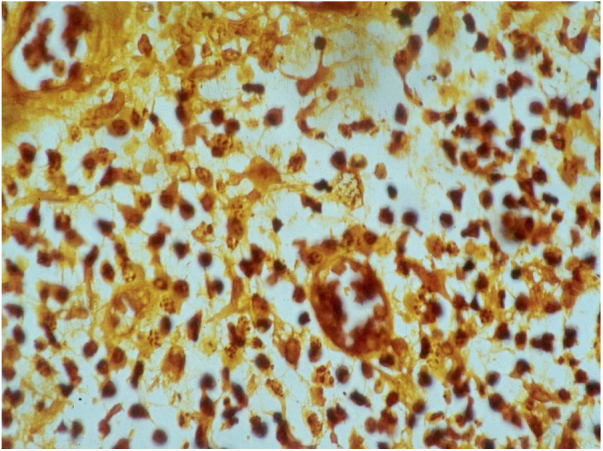
Histopathology showing the presence of Donovan bodies. Warthin-Starry staining (Image credits: Professor Luis Carlos de Lima Ferreira).

The biopsy should be performed preferably on active lesions . The epidermis usually reveals acanthosis with a pseudoepitheliomatous aspect. The dermis shows an intense inflammatory infiltrate consisting mainly of lymphocytes, plasmocytes, histiocytes, and eventually a few eosinophils, with the presence of small neutrophilic abscesses in the epidermis and localized collections of neutrophils in the upper dermis. Histiocytes are usually bulky and vacuolar or cystic in appearance, and in their interior, it is possible to detect the inclusion bodies that are easily identifiable with Giemsa staining.^16^, ^21^, ^27^ The parasite is difficult to see in sections stained with hematoxylin & eosin (Fig. 7). In addition to the anatomopathological examination, transmission electron microscopy can also be used to assess the ultrastructural characteristics of the etiologic agent. However, it is not an exam used in the diagnostic routine due to its high cost and the need for experienced technicians.^21^

Culture of the etiological agent in the yolk sac of a chicken embryo is extremely difficult and is not routinely available due to its high cost, difficulty of execution, and high failure rate. Monolayer cell culture has been described using human monocytes, Hep-2 cells, and mouse peritoneal macrophages.^17^, ^44^ The diagnostic use of polymerase chain reaction (PCR) gene detection techniques is restricted to disease eradication programs, and includes a colorimetric detection method using nucleic acid amplification with *C. granulomatis* primes*.*^45^ Currently, there are no serological tests available for the diagnosis of the disease and, in immunofluorescence tests, fine slices of donovanosis lesions were used as an antigenic source. Studies using these techniques presented good results in patients with old lesions , but the sensitivity was very low in patients with early disease. . The technique was shown to be useful for population studies in endemic areas, but it was not sensitive enough to be considered a confirmatory diagnostic test.^3^, ^21^

## Differential diagnosis

The differential diagnosis of donovanosis lesions should be made with primary syphilis, flat condyloma, phagedenic chancroid, chronic herpetic ulcers, giant acuminate condyloma, lymphogranuloma venereum, and squamous cell carcinoma. It should also include diseases that can present ulcerative-granulomatous lesions, such as cutaneous tuberculosis, paracoccidioidomycosis, and pyoderma gangrenosum. Uncommon and infrequent diseases, such as Langerhans cell histiocytosis and bipolar aphthosis, may also require a differential diagnosis with donovanosis.^16^, ^21^, ^27^

## Treatment

Intravenous emetic tartar, introduced by Aragon and Vianna in 1913, was the first effective drug in the treatment of donovanosis.^7^ With the advent of antibiotics, these became the medication of choice. In difficult to treat patients and in those with scar complications, or in those with significant tissue loss, surgical treatment can also be used as a therapeutic alternative.

Currently, the recommended first-line therapy is azithromycin , two tablets of 500 mg a week for three weeks or until complete healing of the lesions.^3^, ^46^, ^47^ As a second option, doxycycline (100 mg every 12 h) can be used for a minimum period of 21 days or until complete healing. Ciprofloxacin (750 mg every 12 h) for 21 days or until complete healing is also effective. Sulfamethoxazole-trimethoprim (400/800 every 12 h) can also be used, for a period of no less than 21 days.^46^, ^48^, ^49^ If there is no response in the aspect of the lesions in the first days of treatment, it is recommended to add an intravenous aminoglycoside such as gentamicin 1 mg/kg/day, three times a day for at least 21 days. In pregnant patients, erythromycin stearate 500 mg should be used four times a day for at least 21 days.^48^ In refractory lesions or in immunosuppressed or HIV-positive patients, the same therapeutic regimens are suggested, and the use of parenteral therapy with gentamicin or chloramphenicol 500 mg orally, four times a day, should be considered in the most severe cases.^3^, ^27^, ^28^, ^32^^,^^46^, ^48^ Azithromycin (20 mg/kg/day for three consecutive days) should be considered as prophylactic treatment in neonates of mothers with genital lesions of donovanosis.^50^

Regardless of the therapeutic regimen adopted, the duration of treatment should be based primarily on complete healing of the lesions. In the event that scarring does not occur within six weeks, lesion biopsy must be performed to exclude the possibility of malignant transformation, particularly to squamous cell carcinoma. There is no relationship between the size of the lesions and response to treatment. In the rare cases in which relapse occurs after apparent clinical cure, treatment should preferably be repeated with a drug with an antimicrobial spectrum different from that used for primary infection and for a longer period than the first time. After treatment and complete healing of the lesions, the patient must be followed-up for at least one year, every two or three months.

## Complications and sequelae

Among the most common complications are bleeding, genital lymphedema (more frequent in female patients), deforming, and unsightly mutilations and scarring. The possibility of degeneration to squamous cell carcinoma should always be considered, particularly in cases with long evolution. If it is not diagnosed and treated in a timely manner, it can lead to metastases to nearby organs and put the patient's life at risk. Other complications that can affect these patients are scar adhesion of the scrotum to the penis, destruction of the penis body, and stenosis of the urethral, vaginal, and anal orifices. On exceptional occasions, especially in immunosuppressed patients, hematogenous dissemination and involvement of the liver, spleen, and bone may occur, with osteolytic lesions.

## Financial support

None declared.

## Authors’ contributions

Walter Belda Junior: Elaboration and writing of the manuscript.

## Conflicts of interest

None declared.

## CME Questions

**Table:** 



1. Regarding the etiological agent of donovanosis, check the CORRECT statement:

a) It is a Gram-positive, anaerobic and extremely mobile and active coccobacillus.

b) They are easily stained with Giemsa or Wright staining.

c) They have major structural differences with Klebisiella spp.

d) In the form of elongated and tortuous bacilli, they measure from 0.02 µm to 0.2 µm in length.



2. Regarding donovanosis, check the INCORRECT statement:

a) Its incidence has shown marked increase in industrialized countries in recent years.

b) The disease is more common in black individuals and in those with poor hygiene habits.

c) This disease is endemic in tropical and sub-tropical countries.

d) It shows no gender predilection and there are no reports of congenital infection.



3. Tick the INCORRECT statement:

a) Cases of perinatal transmission are characterized by lesions predominantly observed in otorhinolaryngological structures.

b) There is no increased risk of HIV contamination in individuals with donovanosis.

c) There are controversies regarding its transmission being exclusively through sexual contact.

d) Its highest incidence is observed in sexually active groups, although there are cases in children and adolescents without sexual activity.



4. Regarding donovanosis, check the INCORRECT statement:

a) The disease presents high infectivity when considering sexual intercourse as a mode of transmission.

b) It is not possible to rule out the diagnosis based on the absence of disease in sexual partners.

c) Its incubation period is very variable, ranging from 15 to 50 days.

d) Genital lesions are very common, being observed in 90% of cases.



5. Check the INCORRECT alternative:

a) The regions most affected in men are the balanoprepucial groove and the region of frenulum.

b) Clinically, the disease is characterized by nodular-ulcerated lesions with very slow growth.

c) Regional lymph node involvement occurs late in the course of the condition and is similar to that found in cases of lymphogranuloma venereum.

d) The presence of visceral lesions is directly linked to the effectiveness of the patient’s immune condition.



6. Tick the INCORRECT statement:

a) The ulcerative clinical forms present a mild inflammatory reaction and their growth is very slow.

b) Ulcerative lesions are painless and bleed very easily, even spontaneously.

c) The disease has the characteristic of autoinoculation, favored by the oozing of exudate from the lesions.

d) The ulcerative-vegetative clinical form occurs exceptionally, generally being very painful and not bleeding easily.



7. Regarding donovanosis, check the INCORRECT statement:

a) The vegetative forms appear as large lesions, with ill-defined edges and a characteristic fetid odor.

b) Esthiomene occurs more frequently in the female genitalia.

c) The onset of squamous cell carcinoma of the vulva can be observed after a long course of illness.

d) Extragenital lesions can be found in the pharynx, joints, lungs, and liver.



8. Check the INCORRECT alternative:

a) Donovanosis has a much more aggressive and intense course during pregnancy.

b) Giemsa stained cytodiagnosis is the fastest and most economical diagnostic method.

c) The biopsy for diagnosis must be performed on small lesions and always in the center of the ulcer, where greater granulation tissue is found.

d) In the Giemsa stained histological examination, Donovan's bodies are seen within bulky histiocytes.



9. Tick the INCORRECT statement:

a) The culture to identify the etiological agent is extremely difficult, using the medium with Hep-2 cells.

b) Genomic identification techniques by PCR are currently used in the diagnostic routine due to their ease of performance and fast results.

c) The serological tests available for diagnosis have low sensitivity and are not considered to be confirmatory diagnostic tests.

d) The main differential diagnoses are with primary syphilis, phagedenic chancroid, and pyoderma gangrenosum.



10. Check the INCORRECT statement:

a) Intravenous emetic tartar remains efficient in the treatment of donovanosis; it should be considered an alternative drug in cases where antibiotic use is impossible.

b) Currently, the drug of choice is azithromycin, at a dose of 1 g/day until the lesions heal.

c) Drugs such as doxycycline or ciprofloxacin can be used as a second choice in treatment, and must be used for at least 21 days.

d) In neonates of mothers with donovanosis lesions, azithromycin should be considered as prophylactic treatment.

**Table:** 

**ANSWERS** **Hereditary epidermolysis bullosa: update on the clinical and genetic aspects. An Bras Dermatol. 2020;95(5): 551-569.**				
1. d	3. d	5. c	7. c	9. d
2. a	4. c	6. b	8. d	10. a
